# Assessment of Ship-Overtaking Situation Based on Swarm Intelligence Improved KDE

**DOI:** 10.1155/2022/7219661

**Published:** 2022-06-01

**Authors:** Han Xue

**Affiliations:** College of Navigation, Jimei University, Xiamen 361021, Fujian, China

## Abstract

This paper proposes a data-driven risk assessment model for ship overtaking based on the particle swarm optimization (PSO) improved kernel density estimation (KDE). By minimizing the mean square error between the real probability distribution of the ship overtaking point and the kernel density estimation probability distribution calculated by the current kernel density bandwidth, the longitude and latitude of the ship overtaking point are displayed by the color corresponding to the probability as the cost objective function of the search bandwidth of the algorithm. This can better show the distribution of the overtaking points of channel propagation traffic flow. A probability-based ship-overtaking risk evaluation model is developed through the bandwidth and density analysis optimized by an intelligent algorithm. In order to speed up searching the optimal variable width of the kernel density estimator for ship encountering positions, an improved adaptive variable-width kernel density estimator is proposed. The latter reduces the risk of too smooth probability density estimation phenomenon. Its convergence is proved. Finally, the model can efficiently evaluate the risk status of ship overtaking and provide navigational auxiliary decision support for pilots.

## 1. Introduction

### 1.1. Background of the Study

With the development of the shipping industry and the increase in ship types, the traffic flow becomes more dense, the difference in sailing ships speed becomes larger, and the frequency of overtaking behavior between ships gradually increases. In the face of the frequent occurrence of overtaking behavior, relevant provisions, such as the 1972 international maritime collision avoidance rules, provide a comprehensive definition of overtaking behavior, and the maritime department gives many detailed provisions on the ship-overtaking behavior. Overtaking is prohibited or restricted in some waters, especially for key ships, such as dangerous goods ships, passenger ships, passenger rollers, and other ships that need key monitoring. When the ship enters the prohibited overtaking waters of navigation management, it is more necessary for the nearshore vessel traffic management system (VTS) to clarify the ship dynamics at all times, provide efficient command and guidance for the ship, avoid ship collision, and improve the safety of ship navigation [1, 2].

### 1.2. Related Work

In recent years, the Kernel Density Estimation (KDE) has been used in data visualization and hotspot areas. The visualization method of ship trajectories based on KDE can intuitively express the spatial and temporal distribution characteristics of marine traffic. It also indicates the navigation-intensive areas of the channel and the navigation high-risk areas in the ship trajectories data. Several studies have been recently conducted in the KDE area. For instance, Huang et al. [3] proposed a trajectory estimation method based on navigation experience. Liu and Li [4] designed an automatic recognition method of marine traffic flow with AIS Data. Wu et al. [5] designed a variable bandwidth selector in multivariate KDE.

The accuracy of the density kernel estimation method is affected by the bandwidth. However, its practical application is limited due to the time-consuming computation. For instance, Kerm [6] proposed an adaptive KDE algorithm. Xu et al. [7] designed a screened Poisson surface reconstruction using adaptive KDE. Li [8] designed a data-based optimal bandwidth for KDE. Ke and Louani [9] studied the optimal bandwidth selection in KDE. Jim [10] proposed an improved kernel density estimator with adaptive variable bandwidth.

PSO is an iterative optimization algorithm. Zhan et al. [11] provided a survey on evolutionary computation for complex continuous optimization. Li et al. [12] proposed a meta-knowledge transfer-based differential evolution for multitask optimization. Georgiadou and Oriogun [13] proposed a solution encoding scheme in PSO for large-scale optimization. Li et al. [14] designed a pipeline-based parallel particle swarm optimization. Xia et al. [15] proposed the triple archives PSO. Zhan et al. [16] designed an adaptive distributed differential evolution. Liu et al. [17] proposed the coevolutionary PSO with bottleneck objective learning strategy for many-objective optimization. Zhan et al. [18] proposed a heterogeneous differential evolution algorithm and studied its distributed cloud version. SCA is a novel nature like optimization algorithm proposed by Mirjalili in 2016 [19]. Dash et al. [20] made short-term solar power forecasting using hybrid minimum variance expanded RVFLN and Sine-Cosine Levy Flight PSO algorithm.

In this paper, an improved adaptive variable-width kernel density estimator is proposed. It can reduce the risk of too smooth probability density estimation phenomenon, improves the estimation accuracy of probability density, and speed up searching the optimal variable width of the kernel density estimator.

In the area of ship-overtaking possibility, Öztürk proposed a tangible visual analysis tool to analyze maritime traffic on a spatio-temporal basis using AIS data [21]. It helped to provide a better understanding of the macrosecurity structure of the channel and the evidence of individual ships at the microlevel. Korçaka evaluated the collision probability of the risk caused by the Istanbul Strait and the schedule change as a risk reduction option [22]. A credal probability graphical network model based on imprecise probability was established to represent expert knowledge and probabilistic reasoning under uncertainty [23]. Chen used a probabilistic method to assess and reduce the risk of maritime spent nuclear fuel transportation [24]. The event tree was constructed to describe, in detail, the collision process leading to the damage of the transport bucket. Based on the Poisson distribution, the parallel collision probability and cross-collision probability were derived. Du extracted the feature points from the ship AIS data, where the ship behavior was modeled by the track clustering algorithm [25]. The cluster tree was pruned by the appropriate pruning method to realize the track classification and to obtain the ship track features. According to the characteristics of ship traffic flow distribution on each route, the ship collision was simulated by Monte Carlo using the IWRAP theory, where the collision probability of each route was calculated.

The traditional judgment of ship-overtaking situation is mainly based on the definition of overtaking in collision avoidance rules, while only considering the ship's relative orientation, course angle, and speed information, as well as quantitatively dividing the overtaking behavior. The considered factors are too simple. However, in reality, the distance threshold between two ships to judge whether to overtake is affected by several factors such as the hydrological information, meteorological information, nearby traffic flow density and ship maneuverability, ship speed, and ship size.

### 1.3. Contributions

1. The convergence of the proposed algorithm is proved.
2. The proposed algorithm searches the optimal bandwidth of kernel density estimator for ship encountering distribution.
3. A data-driven risk assessment model for ship overtaking is proposed. Considering the analysis of ship-overtaking behavior in channel as the main line, a probability-based ship-overtaking risk evaluation model is developed through the bandwidth and density analysis optimized by intelligent algorithm. The model can efficiently evaluate the risk status of ship overtaking and provide navigational auxiliary decision support for pilots.

## 2. Related Work

### 2.1. Ship-Overtaking Model

**Figure 1:**
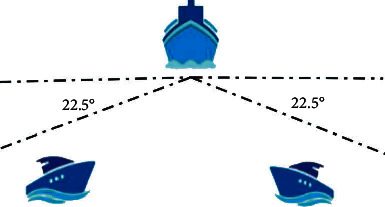
The traditional ship-overtaking model.

The traditional model of ship-overtaking situation is presented in Figure 1.

The overtaking condition is that a give-way ship is greater than 22.5° abaft the beam of the stand-on ship and is within the radian range of the tail light of the stand-on ship. The speed of the give-way ship is greater than that of the stand-on ship.

The distance threshold in the traditional overtaking model is denoted by *D*_*m*_. In general, its value is equal to 3 nautical miles. When the distance between two ships is greater than *D*_*m*_, the overtaking probability is 0. When the distance between two ships is less than *D*_*m*_, the overtaking probability is 1.

When calculating the number of overtaking collisions, the formula of relative velocity is

(1) $$\begin{array}{c} V_{ij}=V_{i}^{\left(1\right)}-V_{j}^{\left(2\right)}, \end{array}$$

where *y* represents the distance between two ships when overtaking and *B* is the width of a given class of ships. *P*_*ij*_ refers to the collision probability of two ships with class *i* and *j*, respectively, if avoidance manipulation is not taken in case of collision.

(2) $$\begin{array}{c} P_{i,j}\left(\text{overtaking}\right)=P\left[y_{i}^{\left(1\right)}-y_{j}^{\left(1\right)}≺\frac{B_{i}^{\left(1\right)}+B_{j}^{\left(1\right)}}{2}\right]-P\left[y_{i}^{\left(1\right)}-y_{j}^{\left(2\right)}≺-\frac{B_{i}^{\left(1\right)}+B_{j}^{\left(1\right)}}{2}\right]. \end{array}$$

In the parallel-route ship-overtaking situation, the ship is sailing along the same course, the ship's course is parallel to each other (the ship's course does not exceed 5), but the two ships have different speeds. In order to estimate the potential collision probability, two parameters should be calculated: overtaking rate (*T*) and the probability of intruding into the ship field when overtaking. Overtaking ratio refers to the number of ships that one ship overtakes another in a parallel route, regardless of the overtaking distance. The overtaking ratio is not the collision frequency unless the water width is zero. When overtaking, the probability of invading the ship field is equal to the event probability that the overtaking distance between two ships is less than the adopted value. The overtaking ratio is calculated by the following formula:

(3) $$\begin{array}{c} T=\frac{N^{2}}{2L}E\left[V_{ij}\right], \end{array}$$

where *N* is expected number of ships in the water area, *L* is the length of channel, and *E*[*V*_*ij*_] is the expected relative speed of all ships of types *i* and *j*.

### 2.2. Nonparametric Multivariate KDE

The multivariate KDE with kernel function *K* and bandwidth *h* is given by [26]

(4) $$\begin{array}{c} \hat{f}=\frac{1}{nh^{d}}\sum_{i=1}^{n}K\left(\frac{x-Y_{i}}{h}\right), \end{array}$$

where *i* represents the accumulated serial number, *n* denotes the total number of samples, *Y* is the give-way ship position of each sample, *Y*_1_,..., *Y*_*n*_ comes from the independent sample with probability distribution of give-way ship position, and *d* denotes the dimension of the ship position data.

*K*(*x*) defined for *d*-dimensional position *x* satisfies the following:

(5) $$\begin{array}{c} \int K\left(x\right)\mathrm{d}x=1. \end{array}$$

For instance, *K* uses the standard multivariate normal density function:

(6) $$\begin{array}{c} K\left(x\right)=\frac{1}{{\left(2\pi \right)}^{d/2}}e^{-x^{T}x/2}. \end{array}$$

The objective is to minimize the mean square error between the true probability distribution of the give-way ship position *f* and the estimated probability distribution of the nuclear density $\hat{f}$ calculated by its current nuclear density bandwidth:

(7) $$\begin{array}{c} \min\text{MISE}=E\iint {\left[f\left(x,y\right)-\hat{f}\left(x,y\right)\right]}^{2}\text{dxdy}. \end{array}$$

## 3. Main Results

The kernel density estimation of the bandwidth is optimized using an intelligent algorithm. MISE is used as the cost objective function of the search bandwidth of the algorithm. Using the color corresponding to probability in order to display the longitude and latitude of the ship encounter, can better display the distribution of the ship traffic flow encounter points, and obtain the reference data of main areas of concern, main situation and collision avoidance distance.

**Figure 2:**
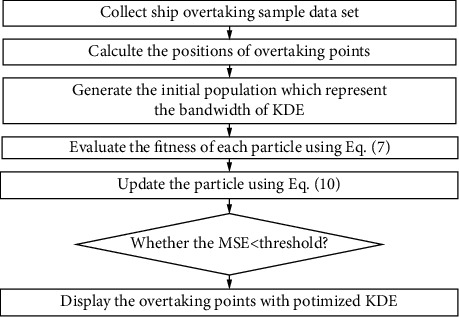
Principle of the proposed algorithm for KDE.

The principle of the proposed algorithm for KDE is shown in Figure 2.

### 3.1. Improved PSO

The position and speed of individuals in the PSO are updated as

(8) $$\begin{array}{c} v_{i,k+1}=v_{i,k}+c_{1}\cos\left(c_{1}\right)r_{1}\left(P_{g,k}-h_{i,t}\right)+c_{2}\sin\left(c_{2}\right)r_{2}\left(g_{k}-h_{i,k}\right),\\ h_{i,k+1}=h_{i,k}+v_{i,k}, \end{array}$$

where *h*_*i*,*k*_ is the position of the *i*th particle in the *k*th iteration, *v*_*i*,*k*_ is the velocity of the *i*th particle in the *k*th iteration, *g*_*k*_ is the best solution obtained up to the *k*th iteration, and *P*_*i*,*k*_ is the best solution obtained by the *i*th particle up to the *k*th iteration.

The quasi-reflection operation ${\hat{h}}_{i,k}$ is

(9) $$\begin{array}{c} {\hat{h}}_{i,k}=\frac{a_{i}+b_{i}}{2}-h_{i,k}, \end{array}$$

where *h*_*i*,*k*_ ∈ [*a*_*i*_, *b*_*i*_], *a*_*i*_ denotes the lower limit of *h*_*i*_,_*k*_, and *b*_*i*_ represents the upper limit of *h*_*i*_,_*k*_.

### 3.2. Channel Overtaking Assessment

According to different navigation behaviors, the traditional overtaking area is divided into prohibited area, dangerous area, and safety area. The overtaking probabilities in the prohibited and safe areas are 1 and 0, respectively. The overtaking probability in the dangerous area conforms to the probability model of specific distribution.

The length of the dangerous area *l* is a variable. Its probability density function can be computed as

(10) $$\begin{array}{c} f\left(l\right)=\frac{1}{nh^{d}}\sum_{i=1}^{n}K\left(\frac{x-Y_{i}}{h}\right). \end{array}$$

The distance of the give-way ship and the stand-on ship is denoted by *L*. The probability of overtaking at the ship's location is given by

(11) $$\begin{array}{c} \mathrm{Pr}\left(L\right)=\left\{\begin{array}{cc} 1, & 0<L\leq d_{m},\\ 1-\int_{0}^{L-d_{m}}f\left(l\right)\mathrm{d}l, & d_{m}<L\leq {\text{D}}_{m}. \end{array}\right. \end{array}$$

The algorithm optimizes the kernel density estimation of the bandwidth. By minimizing the mean square error between the real probability distribution of the ship overtaking point and the kernel density estimation probability distribution calculated by the current kernel density bandwidth, the longitude and latitude of the ship overtaking point are displayed by the color corresponding to the probability as the cost objective function of the search bandwidth of the algorithm. This can better show the distribution of the overtaking points of channel propagation traffic flow. Therefore, the reference data of main areas of concern, main situations, and collision avoidance distances are obtained.

The ScPSO algorithm of the invention searches for the optimal bandwidth of the kernel density estimator, which not only improves the training speed but also the estimation accuracy of the probability density. The principle of the improved KDE-based channel overtaking assessment is summarized as follows:

  Step 1: filter the data in a specific channel from the data of the ship automatic identification system AIS.
  Step 2: identify the overtaking relationship between ships. Based on the judgment of the movement trend between the ships, a specific judgment on whether overtaking occurs between the ships is made, so as to determine the overtaking ship and the chased ship, and extract the data of the overtaking ship and the chased ship from the database. The judgment of overtaking relationship between the ships consists in comparing the relative speed and direction of two ships based on the judgment of the trend of the relative distance between them. Based on the collected AIS data, the ship-overtaking identification method is explored. The dynamic data of overtaking ship and chased ship are obtained, and the method of extracting ship-overtaking data from AIS message data is performed by ship classification, ship time division, judgment of ship relative motion trend, ship distance comparison, ship speed comparison, heading angle judgment, and track verification.
  Step 3: the overtaking probabilities in the prohibited and safe areas are 1 and 0, respectively.
  Step 4: the overtaking probability in the dangerous area conforms to the probability model of specific distribution. For the location points of the dangerous area, the initial bandwidth of the density distribution is generated as the initial population of the particle swarm. Each particle corresponds to an initial nuclear density bandwidth.
  Step 5: calculate MISE.
  Step 6: update the optimal local solution.
  Step 7: update the optimal global solution and gradually approach the optimal bandwidth.
  Step 8: repeat steps 5–7 until the termination condition is met.
  Step 9: on the chart, estimate the probability distribution according to the calculated nuclear density for each ship-overtaking point in the dangerous area and the corresponding final output bandwidth and display it according to the color corresponding to the probability value.

## 4. Case Studies

The ship behavior data are obtained by preprocessing the collected AIS data in 2021. By the judgment method of overtaking relationship between ships, the AIS data of overtaking ship and chased ship are then obtained, which provides complete ship data support for ship-overtaking behavior analysis and overtaking modeling. The AIS data preprocessing process includes AIS data analysis, AIS data cleaning, ship data separation, and ship data classification. The ship-overtaking data acquisition includes the intership motion situation judgment, intership-overtaking relationship identification, and overtaking ship track verification. The intership-overtaking relationship identification includes the distance judgment, ship speed comparison, and heading angle comparison.

The AIS data sample used in the experiment is listed in Table 1.

### 4.1. Case 1: Haicang Channel

The Xiamen Port Haicang channel expansion phase IV Project (E′-17# berth segment) is almost 9.56 km long from the vicinity of Haicang channel e' to Haicang 17# berth. The *E*′-13# berth section of Haicang channel should be constructed according to the requirements of double-track navigation of 200000-ton container ships and 20000-ton container ships, with an operating draft of 15.5 m. It should also meet the requirements of intersection and navigation of 150000-ton container ships and 50000-ton container ships. The Haicang 13#–17# berth section should be constructed according to the requirements of single-track navigation of 200000-ton container ships with an operating draft of 15.5 m.  Table 2 presents the Haicang channel elements.

**Figure 3:**
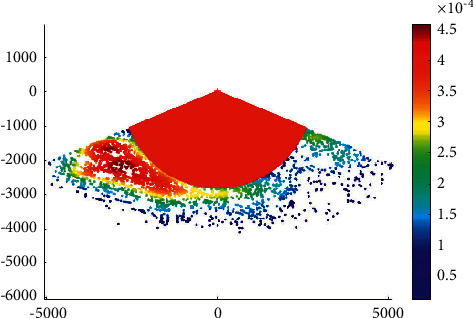
Overtaking ship probability distribution in the Haicang channel.

The overtaking ship probability distribution in the Haicang channel is shown in Figure 3.

It can be seen that the probability of overtaking on the port side is higher than that on the starboard side in the Haicang channel. This is coherent with the ship routing system in Xiamen waters. According to the ship routing system in Xiamen waters, when a ship overtakes another ship in the deepwater route, it should overtake from its port side after obtaining the consent of the overtaken ship.

### 4.2. Case 2: Main Channel

**Figure 4:**
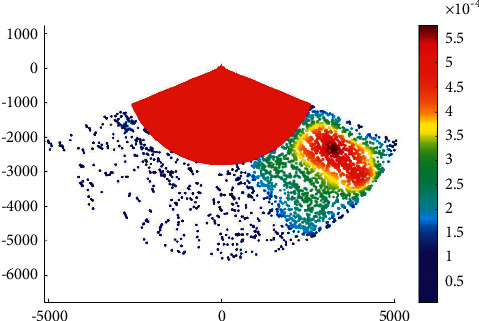
Overtaking ship probability distribution in the main channel.

The phase IV expansion project of the main channel of Xiamen Port starts from the point a' near the 20 m isobath of Xiamen Bay mouth, then passes through the Qingyu waterway and the south water area of Gulangyu Island to the E′ of the Haicang channel mouth of the main channel, with a total length of almost 34.8 km. According to the construction of 200000-ton channel, the whole channel meets the full-tide double-track navigation requirements of 200000-ton container ships and 150000-ton container ships, with an operating draft of 15.5 m and a navigation water level of 0.72 M. It also meets the double-track navigation requirements of 200000-ton container ships and 200000-ton bulk carriers with an operating draft of 15.5 m (the bulk carrier with a full load of 200000 tons should sail by the tide, the tide level is almost 3.9 m, the tide lasts for 4 hours, and the guarantee rate is 90%). The C and c-c1 segments can meet the full-tide double-track navigation requirements of 200000 DWT container ships, with an operating draft of 15.5 m.  Figure 4 presents the overtaking ship probability distribution in the main channel.

The water area within point *C* along the main channel of Xiamen port has a dense distribution of ship-overtaking events along each channel, which is a frequent area of ship-overtaking events. The clear overtaking trend of ships in the channel in the bay is mainly due to the high possibility of ships leaving or entering the port for acceleration or deceleration, which results in frequent overtaking events.

The complexity of the ship navigation environment determines that the single risk evaluation threshold in a certain water area is not suitable for waterway transportation at the present stage, cannot ensure the navigation safety, and is not suitable for intelligent navigation. Therefore, it is important to study the risk evaluation threshold of ship encounter situation under different water conditions in order to ensure the maritime traffic safety and ship collision avoidance.

### 4.3. Case 3: Liuwudian Channel

**Figure 5:**
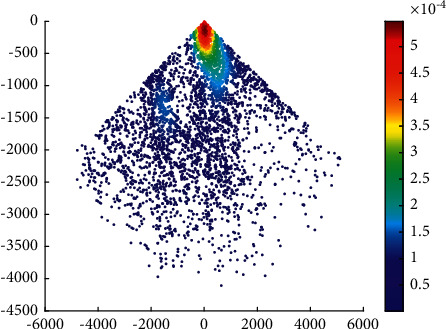
Overtaking ship probability distribution in the Liuwudian channel.

Liuwudian channel is located in the eastern sea area of Xiamen Island. It is the branch channel of the eastern section of the main channel of Xiamen Bay to and from Xiang'an port area. It starts from Xiamen Bay No. 201 light floating near Xiamen Bay No. 9 light floating, passes through the waterway between Dadan Island and Huzi Island, and goes along the waterway on the east side of Xiamen to Xiang'an port area. The total voyage of the channel is about 25.0 km. It can meet the one-way navigation of 70000-ton bulk cargo ships or 100000-ton container ships by the tide.  Figure 5 presents the overtaking ship probability distribution in the Liuwudian channel.

### 4.4. Discussion

#### 4.4.1. Discussion with Bandwidth

**Figure 6:**
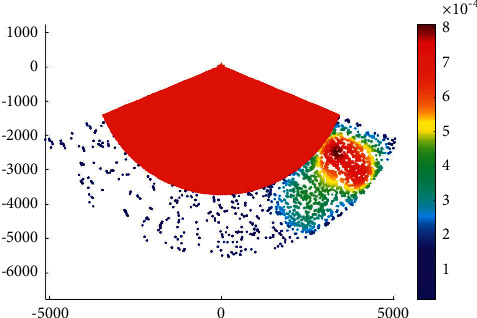
Overtaking ship probability distribution in the main channel with a KDE bandwidth of 300.

Figure 6 shows the overtaking ship probability distribution in the main channel with a KDE bandwidth of 300.

**Figure 7:**
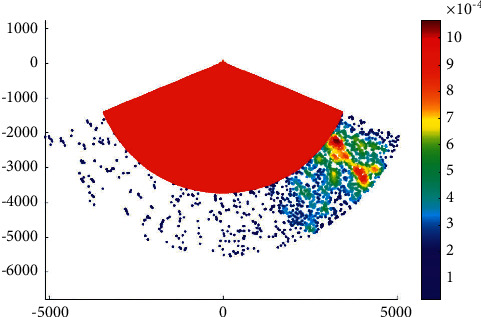
Overtaking ship probability distribution in the main channel with a KDE bandwidth of 100.

Figure 7 presents the overtaking ship probability distribution in the main channel with a KDE bandwidth of 100.

**Figure 8:**
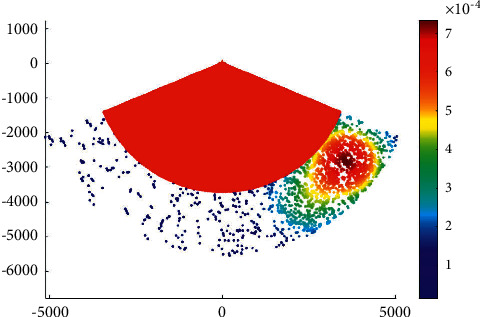
Overtaking ship probability distribution in the main channel with a KDE bandwidth of 600.

Figure 8 shows the overtaking ship probability distribution in the main channel with a KDE bandwidth of 600.

It can be observed that a smaller bandwidth can make more high- or low-value regions appear in the results of overtaking probability distribution, which is suitable for revealing the local characteristics of overtaking probability distribution. In addition, a larger bandwidth can make the hot area of overtaking probability distribution more clear in the global range.

#### 4.4.2. Discussion with Ship Type

**Figure 9:**
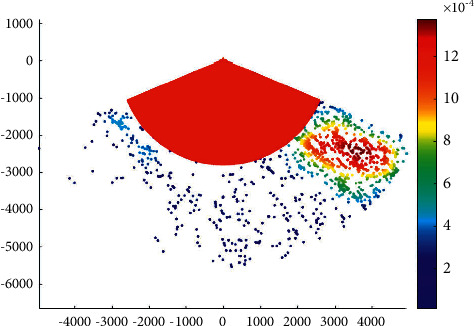
Overtaking ship probability distribution of cargo ships in the main channel.

Figure 9 presents the overtaking ship probability distribution of cargo ships in the main channel.

**Figure 10:**
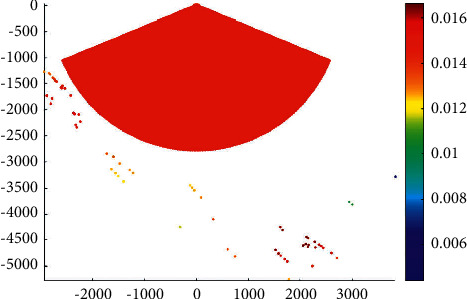
Overtaking ship probability distribution of passenger ships in the main channel.

Figure 10 shows the overtaking ship probability distribution of passenger ships in the main channel.

**Figure 11:**
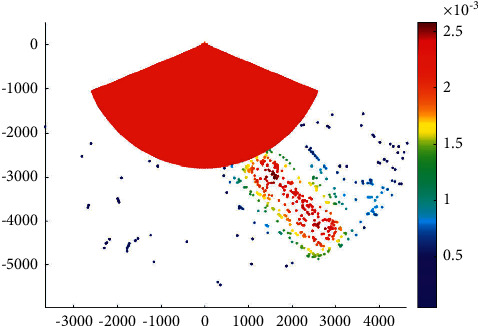
Overtaking ship probability distribution of ocean liners in the main channel.

Figure 11 presents the overtaking ship probability distribution of ocean liners in the main channel.

It can be seen that

1. The frequency of overtaking events of passenger ships is higher than that of other types of ships, while the part with high frequency can reach more than 0.016.
2. Cruise ships are mainly distributed on the main routes of marine transportation. Therefore, the overtaking course is more regular. The part with high frequency can reach almost 0.0025.
3. The overtaking points and overtaking positions of cargo ships are relatively rare. The part with high frequency can reach almost 0.0014. As the traffic flow of cargo ships is in the whole water area, the overtaking course is relatively scattered.

#### 4.4.3. Discussion with *d*_*m*_

**Figure 12:**
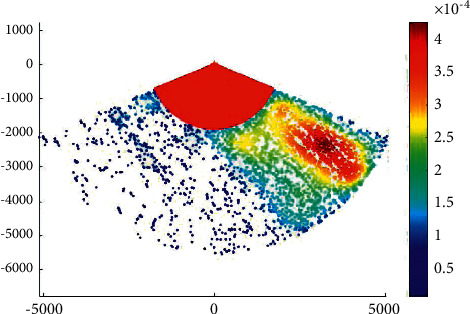
Overtaking ship probability distribution in the main channel when *d*_*m*_ = 1.

Figure 12 shows the overtaking ship probability distribution in the main channel when *d*_*m*_ = 1.

**Figure 13:**
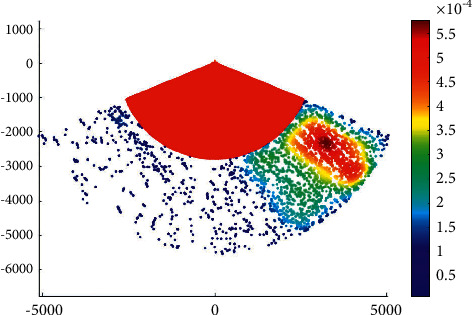
Overtaking ship probability distribution in the main channel when *d*_*m*_ = 1.5.

Figure 13 presents the overtaking ship probability distribution in the main channel when *d*_*m*_ = 1.5.

**Figure 14:**
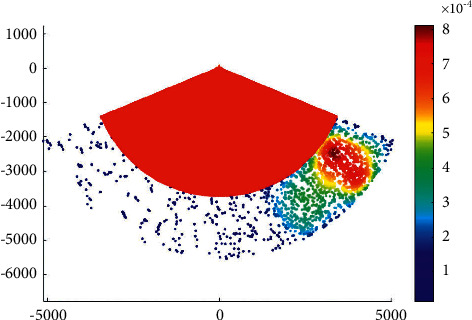
Overtaking ship probability distribution in the main channel when *d*_*m*_ = 2.

Figure 14 shows the overtaking ship probability distribution in the main channel when *d*_*m*_ = 2.

**Figure 15:**
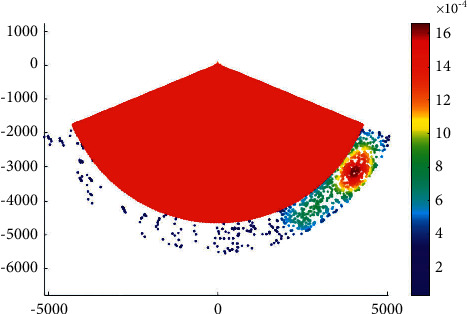
Overtaking ship probability distribution in the main channel when *d*_*m*_ = 2.5.

Figure 15 presents the overtaking ship probability distribution in the main channel when *d*_*m*_ = 2.5.

The distance threshold *d*_*m*_ depends on the constraints of ship type, transverse distance, ship width, distance situation, overtaking situation duration, distance, relative azimuth, course angle, and other factors on the overtaking behavior. In addition, the overtaking process usually starts to show the overtaking situation when it is close to the overtaking ship. This distance is 3 nautical miles less than the visible range of the tail lights of other ships, defined by overtaking. It is the distance threshold in the spatial constraint factors, which can further reduce the spatial constraint range of the target ship, reduce the computational load, and improve the alarm accuracy. The data processing part of the questionnaire uses the method of mathematical statistics and the calculation of statistics such as the average, variance, and median of the data. It also studies the sample data obtained by the questionnaire and interpolates the unreasonable values in the sample data with the average of other samples. Finally, *d*_*m*_ is set to 2 nm.

#### 4.4.4. Discussion with Ship Velocity

**Figure 16:**
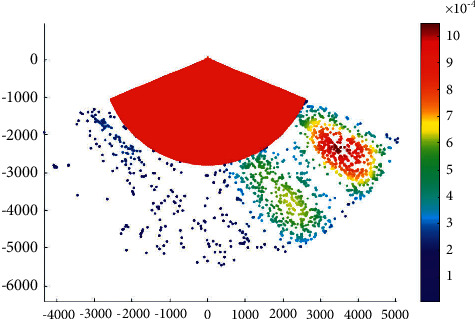
Overtaking ship probability distribution in the main channel when the length of the stand-on ship is less than 3 m.

Figure 16 shows the overtaking ship probability distribution in the main channel when the length of the stand-on ship is less than 3 meters.

**Figure 17:**
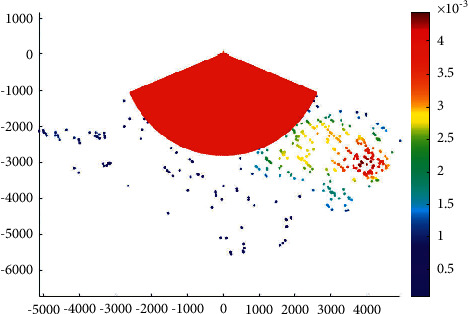
Overtaking ship probability distribution in the main channel when the length of the stand-on ship is between 3 and 6).

Figure 17 presents the overtaking ship probability distribution in the main channel when the length of the stand-on ship is between 3 and 6 meters.

**Figure 18:**
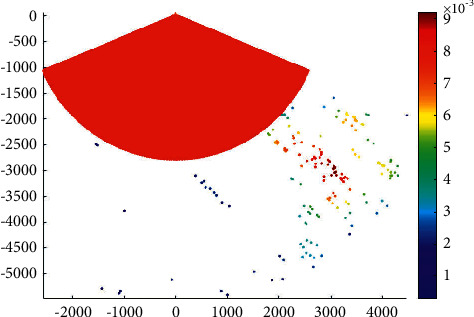
Overtaking ship probability distribution in the main channel when the length of the stand-on ship is greater than 6 m.

Figure 18 shows the overtaking ship probability distribution in the main channel when the length of the stand-on ship is greater than 6 meters.

It can be seen that the smaller the speed of the overtaken ship, the greater the probability of overtaking. During overtaking, the two ships travel in the same direction, with small relative speed and long stalemate time. When the speed difference between the two ships is small, the stalemate time is longer. In this case, the more the factors unfavorable to overtaking, the greater the collision probability. In addition, if the transverse distance between two ships is small, the ship-to-ship effect occurs. If the overtaking ship has a small shore distance, it will produce shallow water effect or bank wall effect. During the stalemate, the ship may be out of control due to the failure of the main engine or steering gear.

### 4.5. Performance Comparison

**Figure 19:**
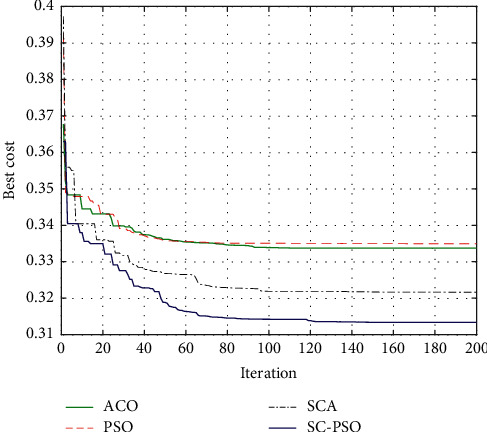
Algorithm convergence comparison.

In this section, the proposed algorithm is compared with PSO, SCA, and ant colony optimization (ACO) as shown in Figure 19.

Figure 19 shows a comparison between the proposed algorithm, SCA, PSO, and ACO.

### 4.6. Time Complexity

**Figure 20:**
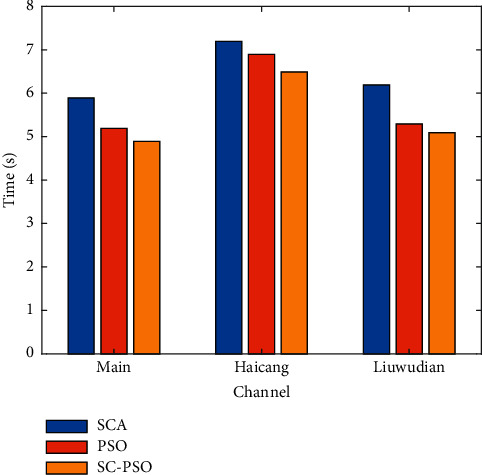
Time-cost comparison.

Figure 20 shows time-cost comparison.

## 5. Conclusion

This paper comprehensively considers the ship's distance, relative azimuth, course angle, speed, ship length, width, transverse distance, distance situation, situation duration, ship type, and other spatial and situation constraints in order to assist traffic managers in the supervision of traffic in the waters under their jurisdiction.

In the process of overtaking, the proposed method correctly grasps the overtaking mode and distance in order to ensure the efficiency of the overtaking action. It first correctly grasps the overtaking mode and speed and obtains the consent of the overtaken ship. It then correctly grasps the safe distance of overtaking while not approaching the overtaken ship too close and prohibiting blocking the bow of the overtaken ship in order to avoid the influence of the force between the hull. Afterwards, the effects of the actions of the chased ship and other factors on the efficiency of the pursuit are considered.

Furthermore, with the environment becoming more complex, the application of other intelligent methods in ship collision avoidance will be tried. We aim at improving the algorithm, like monarch butterfly optimization [27], earthworm optimization algorithm [28], elephant herding optimization [29], moth search algorithm [30], slime mould algorithm [31], hunger games search [32], Runge Kutta optimizer [33], colony predation algorithm [34], and Harris hawks optimization [35].

## Figures and Tables

**Table 1:** AIS data sample.

MMSI	Time	Longitude	Latitude	Course	Speed
200058337	2021-07-01 00 : 33 : 16	118.226127	24.174142	295.0	6.4
200058337	2021-07-01 00 : 48 : 46	118.198253	24.171768	263.0	5.8
200058337	2021-07-01 01 : 18 : 47	118.140987	24.158835	253.0	6.7
200058337	2021-07-01 01 : 35 : 16	118.110263	24.148270	249.0	6.7
200058337	2021-07-01 02 : 16 : 46	118.03285	24.123245	234.0	6.8
200058337	2021-07-01 02 : 17 : 00	118.032075	24.122727	234.2	6.7

**Table 2:** Haicang channel elements.

Point	Latitude	Longitude	Turn angle	Range/m	Course
E′	24°25′46.73″N	118°03′25.03″E	0°0′	3162	286^o^38′08″-106^o^38′08″
E1	24°26′15.83″N	118°01′37.36″E	15°27′	3097	282°59′59″-102°59′59″
E2	24°26′38.13″N	117°59′50.14″E	0°0′	2267	282°59′59″-102°59′59″
E3	24°26′56.10″N	117°58′23.63″E	6°11′	947	289°10′31″∼109°10′31″
E4	24°27′03.64″N	117°57′59.63″E	0°0′	922	289°10′31″∼109°10′31″
E5	24°27′13.37″N	117°57′28.67″E	0°0′	721	289°10′31″∼109°10′31″
E6	24°27′20.98″N	117°57′04.45″E	0°0′	200	289°10′31″∼109°10′31″
E7	24°27′23.10″N	117°56′57.72″E	0°0′	373	289°10′31″∼109°10′31″

## Data Availability

All data, models, and code generated or used during the study appear in the submitted article.
